# Advances in the investigation of the role of autophagy in the etiology of chronic obstructive pulmonary disease: A review

**DOI:** 10.1097/MD.0000000000036390

**Published:** 2023-11-24

**Authors:** Qianxinhong Wang, Wenlong Su, Junnan Liu, Dongkai Zhao

**Affiliations:** a College of Traditional Chinese Medicine, Changchun University of Chinese Medicine, Changchun, China; b College of Pharmacy, Changchun University of Chinese Medicine, Changchun, China; c The Third Clinical Hospital of Changchun University of Traditional Chinese Medicine, Changchun, China.

**Keywords:** autophagy, COPD, etiology

## Abstract

Chronic obstructive pulmonary disease (COPD) is a common chronic respiratory illness. It arises from emphysema and chronic bronchitis and is characterized by progressive and irreversible airflow limitation and chronic inflammation of the lungs, which eventually progresses to pulmonary hypertension, chronic pulmonary heart disease and respiratory failure. Autophagy is a highly conserved cellular homeostasis maintenance mechanism that involves the transport of damaged organelles and proteins to lysosomes for destruction. Dysregulation of autophagy is one of the pathogenic mechanisms of many diseases and is strongly associated with the development of COPD, although the precise mechanisms are unknown. In this paper, we focus on macroautophagy, a type of autophagy that has been thoroughly studied, and describe the characteristics, processes, regulatory pathways, and functions of autophagy, and discuss its relationship with COPD from the perspectives of inflammation, emphysema, mucus hypersecretion, cilia structure and function, airway remodeling, vascular remodeling, and bacterial infections, with a view to searching for the therapeutic targets of COPD from the perspective of autophagy, which is hoped to be helpful for the clinical treatment.

## 1. Introduction

Chronic obstructive pulmonary disease (COPD) is a chronic disease of the respiratory system characterized by persistent respiratory symptoms and airflow limitation that develops in a progressive manner,^[1,2]^ early manifestations of COPD are shortness of breath, dyspnea, cough, and sputum. In advanced stages, COPD can progress to hypoxic respiratory failure and/or chronic hypoxemia.^[3]^ Chronic inflammatory cell infiltration, small airway remodeling, and cupular cell proliferation are the primary pathological changes.^[4]^ Lesions in the lung parenchyma, airways, and pulmonary vasculature have been observed in a substantial number of COPD patients who do not have hypoxia.^[5]^ Statistics show that 3.17 million individuals died from COPD-related causes in 2015.^[6]^ Globally, it is currently the third most common cause of mortality, and is expected to overtake it by 2020. The burden of COPD is significant for both patients and the community. Smoking is a major risk factor for COPD,^[7,8]^air pollution, and biomass fuel exposure. COPD is caused by a combination of intrinsic (e.g., genes) and environmental (e.g., smoking) variables, and up to 50% of smokers develop COPD.^[9,10]^ High levels of oxidative stress and inflammation in the lungs of COPD patients frequently result in airway blockage and loss of the lung parenchyma, which are commonly associated with chronic illnesses such as diabetes, cardiovascular disease, asthma, cancer, and skeletal muscle damage. Pharmacologic and nonpharmacologic therapies are currently available, with pharmacologic therapy comprising bronchodilators, hormones, and combinations of the two. Patient management, home oxygen therapy, home noninvasive ventilation, respiratory rehabilitation, interventional therapy, vaccinations, and surgical therapies are examples of nonpharmacological interventions.

Autophagy is a catabolic process that was initially described in mammals in 1950, in which misfolded or undesired proteins and damaged organelles are delivered to the lysosome and destroyed, which can occur in response to a number of cellular stressors, including hypoxia, hunger, and DNA damage.^[11–13]^ Autophagy is initiated by the induction of several autophagy genes, such as those encoding microtubule-associated protein light chain 3 (LC3), Beclin-1, and other autophagy-associated proteins, all of which play important roles in maintaining intracellular homeostasis under physiological and pathological conditions.^[14–16]^ There are about 40 autophagy-related genes (Atg) work together to promote autophagosome formation.^[17,18]^ Autophagy is also regarded as a protective mechanism against cell death, as it maintains cellular integrity through metabolic precursor renewal and removal of subcellular waste.^[19]^ The formation of double-membrane autophagic vesicles or autophagosomes, which surround damaged cytoplasmic elements, organelles (e.g., mitochondria, endoplasmic reticulum, etc.), or proteins, which then bind to the lysosome to form autophagic lysosomes, is one of the most important features of autophagy. In eukaryotic cells, autophagy is classified into 3 types: macroautophagy, microautophagy, and chaperone-mediated autophagy. Macroautophagy (the term autophagy in this review refers to macroautophagy) is the most commonly studied. Macroautophagy is classified into selective and nonselective autophagy based on cargo specificity and delivery mechanisms. Selective autophagy uses autophagy to selectively breakdown mitochondria, other organelles, bacteria, and protein aggregates.^[20,21]^ Autophagy has emerged as an important cellular process involved in cellular homeostasis and survival mechanisms in recent years, and it plays a vital role in cell physiology, energy metabolism, the innate immune system, the adaptive immune system, and programmed cell death.

Increasing evidence suggests that the onset of COPD is linked to dysregulated autophagy, and the current literature on the association between COPD and autophagy is perplexing. Therefore, in this paper, we will discuss the critical role of autophagy in COPD from multiple perspectives of several hotspot pathogenic mechanisms of COPD: chronic inflammation, oxidative stress, emphysema, mucus hypersecretion, airway remodeling, and bacterial infections, with the goal of providing a theoretical foundation for clinical COPD treatment targeting autophagy.

## 2. Autophagy

### 2.1. The process of autophagy

**Figure 1.**
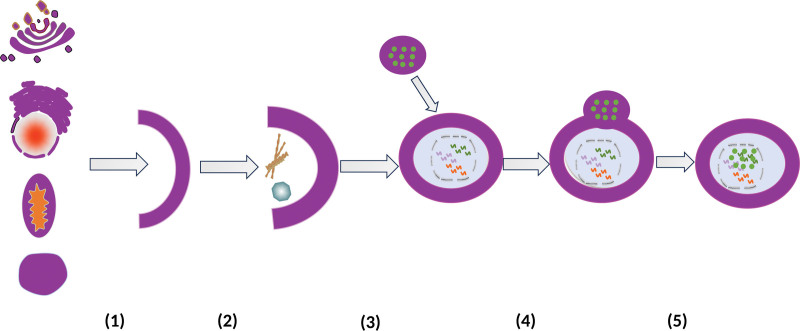
The process of autophagy. (1) Isolated membranes from sources such as the endoplasmic reticulum, Golgi apparatus, mitochondrial outer membrane, and plasma membrane (2) Separation of cytoplasmic proteins and organelles to be degraded in membrane-enclosed cells (3) Formation of double-membrane circular vesicle structures – “autophagosomes,” fusion of “autophagosomes” with lysosomes (4) “Autolysosome” formation (5) Cytoplasmic components are degraded by a range of lysosomal degrading enzymes.

Autophagy is a highly active process that is separated into stages: (1) Endoplasmic reticulum, Golgi apparatus, outer mitochondrial membrane, and plasma membrane isolated membranes surround cytoplasmic proteins and organelles to be destroyed in the cell, generating double-membrane circular vesicles known as “autophagosomes”^[22–24]^; (2)Autophagosomes fuse with lysosomes to generate “autolysosomes,” and the contents of these autolysosomes are transferred to lysosomes; (3) A variety of lysosomal degradation enzymes (e.g., histone proteases and other acidic hydrolases) degrade cytoplasmic components to renew metabolic precursor molecules (amino acids, fatty acids); (4)The digested components are discharged into the cytoplasmic lysate, where they might be reused in biosynthetic pathways.^[25,26]^ The process of autophagy is illustrated in Figure 1.

### 2.2. Regulatory pathways of autophagy

Autophagy activation is regulated by 2 signaling pathways: the mammalian target of rapamycin (mTOR)-dependent and mTOR-independent pathways.^[25]^ The phosphatidylinositol 3-kinase/protein kinase B/mTOR signaling pathway plays a key regulatory role in autophagy.^[27]^ mTOR, an evolutionarily conserved serine-threonine kinase, is a sensor of environmental, cellular nutritional, and energetic status, mTOR regulates crosstalk between many cellular processes, including autophagy, translation, inflammation, apoptosis, and energy metabolism. There are 2 types of mTOR complexes, mTORC1 and mTORC2, which can be activated by large amounts of mitogens, nutrients, and growth factors, and regulate a variety of functions, including cell growth, development, proliferation, autophagy, innate immune response, and adaptive immune response.

### 2.3. Functions of autophagy

Selective and nonselective autophagy serve various tasks: selective autophagy targets and eliminates specific subcellular components, whereas nonselective autophagy phagocytoses and destroys intracellular proteins under nutritional deprivation and recycles them.^[28]^ The removal of polyubiquitin protein clumps (aggregated protein phagocytosis), malfunctioning organelles such as mitochondria (mitochondrial phagocytosis), and invading pathogens (xenophagocytosis) are examples of selective autophagy functions.^[15,29–31]^ Mitochondrial autophagy is the selective elimination of mitochondria, which regulates mitochondrial number to match metabolic demands. PINK1 and PARK2 deficiency in mitochondrial autophagy regulator genes causes mitochondrial damage and malfunction, which leads to Parkinson’s disease.^[31]^ Xenophagy can aid the immune response during infection by selectively destroying intracellular invading microorganisms.^[32]^ Many inflammatory disorders are related to xenophagy, which can also play a role in host defense by removing intracellular pathogens and improving immune identification of infected cells through the synthesis of antigenic bacterial peptides.^[33]^ Autophagy also controls lipid metabolism.^[34]^ Selective autophagy is essential for the maintenance of cellular homeostasis.

## 3. COPD

### 3.1. Pathogenic factors of COPD

Although most studies currently consider smoking to be the most important risk factor for COPD, some patients with COPD do not have a history of smoking.^[35]^ Although COPD becomes symptomatic in the 40 to 50 year old age range, its initiation may occur decades before symptoms appear. Environmental factors, such as air pollution, tobacco smoke, poisonous gases, and vehicular exhaust, all contribute to the progression of COPD. Age and sex are other determinants: the older the patient, the higher the probability of COPD death, and older patients are more prone to respiratory failure and expedited mortality.^[36]^ COPD prevalence between men and women have been researched inconsistently, with some studies claiming that males smoke more than women and thus have a higher prevalence of COPD than women, while others claim that women are at a higher risk of acquiring COPD. Adverse childhood environmental factors, bronchial hyperresponsiveness and impaired lung function due to asthma, body mass index, respiratory infections, genetics, poverty, and socioeconomic status are all closely linked to the development of COPD.^[37]^ Shortly after birth, children or low-birth-weight infants whose mothers smoked during pregnancy had lower lung function.^[38]^ Bronchial hyperresponsiveness is a defining feature of asthma; however, it is also present in COPD, which precedes the onset of COPD symptoms, and severe bronchial hyperresponsiveness can accelerate the deterioration of lung function.^[39]^ COPD is a complex disease that is affected by a combination of genes and the environment, and multiple genome-wide association studies have revealed genes related to the presence and severity of airway obstruction.

### 3.2. Pathogenesis of COPD

Chronic inflammation, oxidative stress, protease-antiprotease imbalance, cellular senescence, and mucus hypersecretion are hallmarks of COPD etiology. Chronic inflammation is extensive, including in the peripheral airways and lung parenchyma, with a characteristic inflammatory pattern of increased numbers of T lymphocytes, B lymphocytes, macrophages, and neutrophils, and the inflammatory response worsen during acute exacerbations. In COPD, the inflammatory response involves both intrinsic (neutrophils, macrophages, eosinophils, mast cells, NK cells, T cells, intrinsic lymphocytes, and dendritic cells) and adaptive immunity, as well as structural cell activation (airway and alveolar epithelial cells, endothelial cells, and fibroblasts). Many studies have found that oxidative stress is significantly higher in COPD patients.^[40]^ The lungs are one of the most vulnerable target organs to oxidative stress, and oxidative damage to the lungs can occur as a result of inhalation of hazardous particles and gases from the external environment, as well as endogenous oxidants produced by exposure to local microenvironments.^[41]^ Some reactive oxygen species (ROS) are formed during normal metabolic activities in the human body, and cells express a significant variety of endogenous antioxidants, including superoxide dismutase, catalase, and glutathione peroxidase, to neutralize free radicals and mitigate the detrimental effects of ROS.^[42]^ When the formation of ROS exceeds antioxidant defense mechanisms, oxidative stress occurs, causing damage to lipids, proteins, and DNA. Oxidative stress inhibits the activity of antiproteases such as α1-antitrypsin and increases elastin breakdown in lung parenchyma. ROS directly activates matrix metalloproteinases, causing a protease-antiprotease imbalance that boosts the ability of matrix metalloproteinases to destroy the extracellular matrix and promote lung parenchyma damage.^[43]^

COPD has been demonstrated to be an autophagy-related lung illness, and there is growing evidence that autophagy is dysregulated in COPD patients, with 2 extremes of autophagy dysregulation in COPD patients: increased autophagy and impaired autophagy. Autophagy deficiency causes an inability to remove oxidatively damaged organelles or proteins, which accelerates COPD development.^[44]^ On the other hand, autophagy overactivity causes increased cell death/apoptosis and cilia damage, which leads to emphysema.^[45]^

## 4. Autophagy and COPD

Autophagy has been proven to be both protective and harmful in a variety of animal models, implying that its involvement in human diseases is multifaceted. Electron microscopy revealed an increase in the number of autophagosomes and the expression of autophagy proteins LC3B-II, Atg4, Atg5-12, and Atg7 in lung tissues from COPD patients compared with controls.^[46]^ Increased LC3 expression in alveolar macrophages isolated from smokers is associated with impaired autophagic flow^[47]^; autophagy is similarly elevated in lung tissue of patients with COPD due to α1-trypsin insufficiency in some nonsmokers. In contrast, elevated autophagy markers were not observed in lung tissue from people with other lung conditions, such as systemic sclerosis, tuberculosis, cystic fibrosis, or idiopathic pulmonary fibrosis. These findings show that autophagosome production and autophagy protein expression could be employed as biomarkers for specific lung illnesses.

### 4.1. Autophagy and structural changes in lung tissue

#### 4.1.1. Autophagy and cilia structure and function.

There are 2 types of cilia in the lungs: sensory primary cilia, which are responsible for signaling, and motor cilia, which are responsible for eliminating mucus and waste particles. Patients with COPD have lower clearance of airway mucosal cilia and shorter cilia on airway epithelial cells, making their airways more prone to abnormal inflammatory responses and pathogenic infections.^[48]^ Smoke and other harmful gases shorten the length of the cilia of airway epithelial cells, resulting in severe cell death, followed by epithelialization of the cup cells, which secrete excessive airway mucus, resulting in impaired clearance of airway mucosal cilia and, the aggregation of harmful particles, pathogens, and other pathogens in the respiratory tract, leading to infections.^[49]^

Autophagy regulates the cilia in 2 ways.^[50]^ On the one hand, autophagy can degrade proteins essential for intraflagellar transport, inhibiting ciliogenesis.^[51]^ On the other hand, autophagy can degrade proteins that hinder ciliogenesis and promote ciliogenesis.^[52]^ Cilia length is regulated by autophagy-dependent pathways during cigarette smoke (CS) exposure; CS-induced cilia shortening occurs via an autophagy-dependent mechanism controlled by the deacetylase HDAC6 (histidine deacetylase 6), and autophagy-impaired mice are resistant to CS-induced cilia shortening. CS exposure increases autophagy in airway epithelial cells, causing the degradation of cellular ciliary proteins and ciliary components, culminating in cilia shortening and, in severe cases, ciliated cell loss and death.^[53]^ Mice with defective autophagy prevented CS-induced reduction of mucosal ciliary clearance function.^[54]^

#### 4.1.2. Autophagy and emphysema.

All levels of bronchial tubes and terminal alveolar components in the lungs are referred to as lung parenchyma. Emphysema is a degenerative condition in the lung parenchyma that is characterized by a gradual loss of elasticity of the lung tissues and permanent swelling of alveolar cavities in individuals with COPD. Cigarette smoking is the most prevalent trigger, and repeated exposure to CS induces ROS production, inflammatory oxidative stress, and apoptosis, leading to alveolar space enlargement and emphysema progression.^[55]^ Because CS exposure not only induces COPD emphysema but also lowers the lung’s ability to fight infection,^[56]^ emphysema is linked to acute or recurring chronic infections, which finally leads to respiratory failure.^[57]^ There is no agreement on the association between autophagy and emphysema, with some claiming that inhibiting autophagy can limit the development of emphysema and others believing that defective autophagy causes the development of emphysema.

Some researchers believe that autophagy contributes to emphysema development. Smoking is the most prevalent cause of emphysema and smoke exposure can enhance the generation of mitochondrial ROS in lung epithelial cells, causing autophagy. Autophagy aggravates mitochondrial damage and depolarization by boosting receptor-interacting protein 3 levels and inducing apoptosis in lung epithelial cells.^[58]^

Toll-like receptors (TLRs) have been a growing field of study in recent years, and because TLRs in lung tissue are directly related to the surrounding environment, they play a significant role in responding to air components (oxygen and particulate matter). According to other studies, TLR4 is critical for the maintenance of lung tissue structures. TLR4 loss causes the activation of a new NADPH oxidase (Nox) in the lungs and endothelial cells, which increases oxidant generation and elastolytic activity^[59]^ altering the normal structure of lung tissue and inducing emphysema. TLR are linked to emphysema, and TLR also play a role in autophagy regulation.^[60,61]^ An experimental study by CH concluded that there is a link between TLR4 and autophagy, and in vitro and in vivo experiments using lung tissues from patients with COPD and Tlr4 mutant and Tler4-deficient mice demonstrated that TLR4 inhibits the development of CS-induced emphysema by suppressing autophagy.^[62]^

In a chronic cigarette smoke-induced animal model, the autophagy protein LC3B and its related regulatory molecule Egr-1 increased alveolar lumen widening and, therefore, emphysema progression.^[46,63]^ According to Hou et al,^[64]^ porcine pancreatic elastase induces lung autophagy and promotes lung cell death and emphysema via the MAPK8 and MAPK14 pathways.

Additional investigations have shown that CS affects autophagy, hastens lung aging, and worsens emphysema.^[44]^ CS exposure impairs the intracellular autophagy pathway, leading to the accumulation of intracellular misfolded or polyubiquitinated proteasomes and autophagy intermediates, resulting in an imbalance of intracellular homeostasis, cell lysis, cell death, and ultimately the destruction of lung tissue structure and the development of emphysema.^[44,65,66]^ Using CS-exposed mouse lungs and peripheral lung tissues from COPD patients, Bodas found that sphingolipid lactose ceramide buildup initiates aberrant autophagy (p62 accumulation) and apoptosis, which increases emphysema development.^[67]^

#### 4.1.3. Autophagy and airway remodeling.

Airway remodeling is a pathological feature of COPD progression, manifested by squamous cellular metaplasia of the ciliated columnar epithelium of the pseudocomplex layer of the bronchial mucosa, absence of cilia, reduction, and motility dysfunction, and hyperplasia of the smooth muscle and fibrous connective tissues, accompanied by inflammatory cell infiltration and an increase in the number of cuprocytes. Airway remodeling is a change in the morphology and structure of the trachea that involves several physiological processes in cells (e.g., adaption, proliferation, migration, apoptosis, and extracellular matrix formation and breakdown). Airway remodeling is characterized by pathogenic changes in many types of airway cells (bronchial epithelial cells, airway smooth muscle cells, fibroblasts, etc.) that are intimately associated with autophagy.

Bronchial tube epithelial cells can expel dust, bacteria, and other dangerous elements inhaled from the outside world to the larynx via regular swinging of the cilia, and subsequently out of the body by coughing. Tobacco smoke and other hazardous gases act on bronchial epithelial cells on a chronic basis, generating adaptive alterations and apoptosis. Neutrophil elastase (NE) is a protein hydrolyzing enzyme, the secretion of which is increased by smoke exposure, excessive NE damages bronchial epithelial cells,^[68]^ and NE increases levels of placental growth factor, which stimulates bronchial epithelial cells to increase intracellular autophagic vesicles.^[64]^ As a result, NE initiates intracellular autophagy, and inhibiting this process could be used to treat COPD. TIE LIU grew human bronchial epithelial cells exposed to PM2.5, and found that the levels of autophagy-related proteins LC3B and BECN1, LC3BII/LC3BI, and autophagosome formation were associated with PM2.5.^[69]^

#### 4.1.4. Autophagy and vascular remodeling.

In the early stages of COPD, vascular wall thickening and vascular endothelial cell destruction can be seen. As the disease progresses, vascular smooth muscle cell thickening and increased collagen fibers are observed, along with inflammatory cell infiltration. Several studies have implied that autophagy play a role in vascular remodeling.^[70,71]^ Relevant investigations have revealed that moderate vascular endothelial cell autophagy protects blood arteries; however, whether excessive autophagy causes vascular endothelial cell dysfunction remains unknown.^[71]^

### 4.2. Autophagy and changes in the internal environment of lung tissue

#### 4.2.1. Autophagy and chronic inflammation.

Chronic inflammation, involving both intrinsic and adaptive immunity, is a major contributor to COPD pathogenesis. Following smoke inhalation, harmful components invade the lung tissue, activating pattern recognition receptors, structural cells (alveolar epithelial cells, airway epithelial cells, fibroblasts and endothelial cells), and intrinsic immune cells (neutrophils, macrophages, dendritic cells, natural killer cells, and eosinophils) within the airway, which activate inflammation-associated damage-associated molecular patterns, and a large number of inflammatory mediators, such as cytokines, chemokines, growth factors, oxygen free radicals, acute-phase response proteins and antimicrobial peptides, and enzymes (e.g., proteases), which produce substances that act on the lung tissue and contribute to mucus hypersecretion, destruction of the lung parenchyma and alveolar interstitium, and airflow obstruction.

The levels of inflammation in lung tissue correlate with autophagy levels; CS can cause autophagy and thus COPD, Yunxiao Li’s study^[72]^ with adult male wild-type (WT) C57BL/6J and Ephx2-/- mice suggested that Ephx2 may hold promise as a therapeutic target for CS-induced COPD, and may play a protective role in lung tissue by inhibiting autophagy and resisting inflammation, supporting the idea that autophagy is enhanced and Ephx2 lacks inhibition of autophagy in a mouse model of COPD. Li found that silymarin inhibited autophagy in human bronchial epithelial cells, reducing cigarette smoke extract (CSE)-induced chronic inflammation.^[73]^

mTOR inhibits autophagogenesis.^[74,75]^ According to Wang et al,^[76]^ mTOR may prevent CS-induced inflammation and emphysema by reducing autophagy and apoptosis, indicating that mTOR activation may be a therapeutic target for COPD.

Macrophages play a critical role in the inflammatory responses in COPD. A study of autophagy in alveolar macrophages isolated from the lungs of heavy smokers revealed the activation of autophagic markers, such as autophagosomes and LC3-II production, in smokers’ macrophages; autophagosome production was increased within macrophages, according to ultrastructural analyses.^[47]^

#### 4.2.2. Autophagy and mucus hypersecretion.

One of the pathological hallmarks of COPD is airway mucus hypersecretion and mucin MUC5AC. Excessive mucus secretion obstructs the airway, causing the patient to cough up sputum and produce acute exacerbations. In airway epithelial cells, CSE increased the expression of the mucus gene MUC5AC.^[77]^ Autophagy suppression reduces MUC5AC expression; hence, inhibition of autophagy is a treatment option for COPD.^[78]^

#### 4.2.3. Autophagy and bacterial infection.

Patients with COPD are more likely to develop lower respiratory tract infections, which can result in acute exacerbations and increased hospitalization and mortality rates.^[79]^ Lower respiratory tract infections are characterized by a high prevalence of bacterial and viral infections, which can significantly increase the level of inflammation, degree of impairment of lung function, degree of airflow limitation, and hasten the patient’s deterioration. Streptococcus pneumoniae, Haemophilus influenzae non-typhoidal, and Pseudomonas aeruginosa are the most prevalent bacterial pathogens causing illnesses.^[80–82]^

Macrophages, which control lung infection through phagocytosis to eliminate pathogens, play an important role in the protection of the lung tissue against pathogen infection.^[83,84]^ Autophagy is one of the most important host defense systems against pathogens such as bacteria, viruses, and parasites.^[15,85,86]^ In COPD patients with COPD, alveolar macrophage phagocytosis is reduced, and one probable explanation is CS-induced autophagic damage.^[79,87,88]^ Garrett Pehote conducted in vivo studies with RAW264.7 cells and Pseudomonas aeruginosa (PA01-GFP) to confirm that the mechanism of decreased phagocytosis is CS-induced autophagic damage.^[89]^ CS exposure reduces the expression of TFEB, a key autophagy regulator in RAW cells, affecting the autophagic process, impairing bacterial transport to lysosomes, preventing cells from removing harmful pathogens via phagocytosis, increasing the susceptibility of lung tissues to pathogens, and causing recurrent infections in smokers. Fisetin, an autophagy-mediated antioxidant, was also found to restore phagocytic capabilities.

#### 4.2.4. Autophagy and oxidative stress.

Patients with COPD have an oxidative and antioxidant imbalance, and oxidative stress develops in biological systems when endogenous antioxidant defenses are impaired and/or overwhelmed by ROS.^[90]^ Exogenous factors contributing to oxidative stress injury include smoking, toxic gases, particulate matter, and biofuels, which are structural and inflammatory cells, that trigger oxidative stress injury through NADPH oxidase 2, xanthine/xanthine oxidase, mitochondrial respiration, and heme peroxidase.^[91]^ The high oxygen content of the lung tissue, direct exposure to the external environment, and frequent contact with harmful gases, pathogens, and toxins, among other factors, make it highly susceptible to oxidative stress damage, promoting the production of large amounts of ROS, which in turn causes the onset and progression of COPD.^[92]^ Autophagy is a negative feedback regulatory process that removes ROS from the cells and protects them from oxidative damage.

Dysregulation of autophagy as a result of smoking, environmental damage, or aging, leads to an increase in aggregate formation and the creation of ROS, all of which contribute to the pathogenesis of COPD. Several in vitro studies using low-dose CSE have shown that loss of autophagy enhances smoke-induced epithelial cell senescence, mitochondrial ROS production, and accumulation of ubiquitinated proteins,^[93–95]^ implying that autophagy may have protective effects on epithelial cells in COPD patients.

#### 4.2.5. Autophagy and cellular senescence.

Cellular senescence caused by CS is a pathogenic process in COPD, and autophagy is closely related to cellular senescence. Satoko Fujii’s cellular experiments^[94]^ showed that a decline in autophagy prevents damaged proteins (including ubiquitinated proteins) from being cleaned up in a timely manner, accelerates cellular senescence. However, activating autophagy, particularly selective autophagy, has negative effects on epithelial cells.^[46,58,62,96]^

#### 4.2.6. Autophagy and protease-antiprotease imbalance.

Patients with COPD with α1-antitrypsin deficiency and increased elastase levels.^[97]^ The autophagy marker molecule LC3BII/LC3BI is found in the lung tissues of emphysema patients with α1-antitrypsin deficiency, implying a link between protease-antitrypsin imbalance and autophagy.^[46,98]^ Hou et al^[64]^ demonstrated that excess trypsin induces the expression and secretion of placental growth factor, which up-regulates MAP1LC3B/LC3B and mediates the inactivation of mTOR through the AMPK14/p38alpha MAPK and AMPK8/JNK1 signaling pathways, promoting autophagy and aggravating emphysema, further suggesting that an imbalance of protease and antiprotease causes autophagy to occur and promotes the development of COPD.

## 5. Discussion

Autophagy has both protective (anti-pathogenic) and detrimental (pathogenic) functions. Enhanced autophagy usually has a beneficial effect on cellular function and homeostatic regulation, which improves lung health and lengthens life. However, excessive autophagy may harm the lungs and shorten life expectancy.^[99]^ When autophagy is appropriately triggered and autophagosomes are properly converted to autolysosomes, and correct autophagic pathways remove toxic protein aggregates, regulate mitochondrial populations, and clean up bacteria and viruses, autophagy increases cell survival. Although autophagy can be a pro-survival process, especially during dietary deficiency, its role in disease pathogenesis is potentially flexible and complex, and the activation or inhibition of autophagy could be used to alter human disease progression. Targeted therapeutic approaches can be developed to address the mechanisms by which autophagy affects the development of disease, promoting the formation of effective autophagosomes and the removal of damaged organelles, proteins, or cellular debris. The effects of autophagy are multifaceted; therefore, the pros and cons need to be weighed and used with caution when using autophagy modulators to treat diseases.

## Author contributions

**Conceptualization:** Qianxinhong Wang.

**Data curation:** Qianxinhong Wang.

**Formal analysis:** Wenlong Su.

**Funding acquisition:** Dongkai Zhao.

**Investigation:** Wenlong Su.

**Methodology:** Qianxinhong Wang.

**Project administration:** Dongkai Zhao.

**Resources:** Junnan Liu.

**Software:** Qianxinhong Wang

**Supervision:** Dongkai Zhao.

**Validation:** Qianxinhong Wang.

**Visualization:** Junnan Liu.

**Writing – original draft:** Qianxinhong Wang.

**Writing – review & editing:** Dongkai Zhao.
